# Design and Preparation a New Composite Hydrophilic/Hydrophobic Membrane for Desalination by Pervaporation

**DOI:** 10.3390/membranes13060599

**Published:** 2023-06-13

**Authors:** Tarik Eljaddi, Eric Favre, Denis Roizard

**Affiliations:** LRGP—Laboratoire Réactions et Génie des Procédés, UMR 7274, 54001 Nancy, France

**Keywords:** desalination, CTA composite membranes, thermo-pervaporation, mass transfer prediction, seawater

## Abstract

Herein, experimental and theoretical approaches were used to design a new composite membrane for desalination by pervaporation. The theoretical approaches demonstrate the possibility to reach high mass transfer coefficients quite close to those obtained with conventional porous membranes if two conditions are verified: (i) a dense layer with a low thickness and (ii) a support with a high-water permeability. For this purpose, several membranes with a cellulose triacetate (CTA) polymer were prepared and compared with a hydrophobic membrane prepared in a previous study. The composite membranes were tested for several feed conditions, i.e., pure water, brine and saline water containing a surfactant. The results show that, whatever the tested feed, no wetting occurred during several hours of desalination tests. In addition, a steady flux was obtained together with a very high salt rejection (close to 100%) for the CTA membranes. Lastly, the CTA composite membrane was tested with real seawater without any pretreatment. It was shown that the salt rejection was still very high (close to 99.5%) and that no wetting could be detected for several hours. This investigation opens a new direction to prepare specific and sustainable membranes for desalination by pervaporation.

## 1. Introduction

Nowadays, the industrial challenge of the 21st century is to provide enough drinking water to the global population [1]. The most sustainable resource is obviously seawater, which covers more than 70% of the global surface of the Earth. The main commercial process used in desalination to produce drinking water are currently multistage flash (MSF), multi-effect distillation (MED) and reverse osmosis (RO) [2,3]. According to recent statistics, RO presented 68.7% of the total installed desalination capacity in the last year, with 17.6% for MSF, followed by 6.9% for MED [3,4]. In addition, the annual growth is estimated at ~55%, and the total market of this technology for desalination application was USD 30 billion for 2015 [5]. The drawbacks of RO are (i) the high consumption of energy, since the driven force is based on high pressure that affects the price of water production [6,7] and (ii) the brine rejection in the environment that induces many environmental issues [8]. For overcoming these drawbacks, the emerging technologies are membrane distillation (MD) [9] and pervaporation (PV) [10]. Moreover, it is worth noting that these technologies can be coupled with RO to treat brines that have a high salt concentration [11,12]. MD and PV are both thermal membrane processes, i.e., the water is transported through the membrane in a vapor state, thus giving rise to a high energy demands due to the vaporization of water from the feed side to the permeate side. Thus, MD and PV have a similar energy consumption [13]. The membrane used in MD must be hydrophobic with a porous structure. For this reason, most of the materials used in MD are polytetrafluoroethylene (PTFE), polyvinylidene fluoride (PVDF) and polypropylene (PP) [14]. A detailed description of MD can be found in the literature [15,16]. However, the main drawback of MD is the wetting issue that can occur when the feed liquid water enters the membrane pores. Consequently, the salt rejection is dramatically decreased, and simultaneously, the process loses its selectivity [17]. Contrarily, in PV technology, the membrane must be dense and the nature of the membrane can either be hydrophilic or hydrophobic [18]. PV is now a mature technology that is used at an industrial scale for dehydration, organics removal from aqueous solution, separation of some organic mixtures and concentration of aqueous solutions [19]. Table 1 summarizes the main differences between MD and PV (for more details, the reader can see the review papers cited in this table).

**Figure 1:**
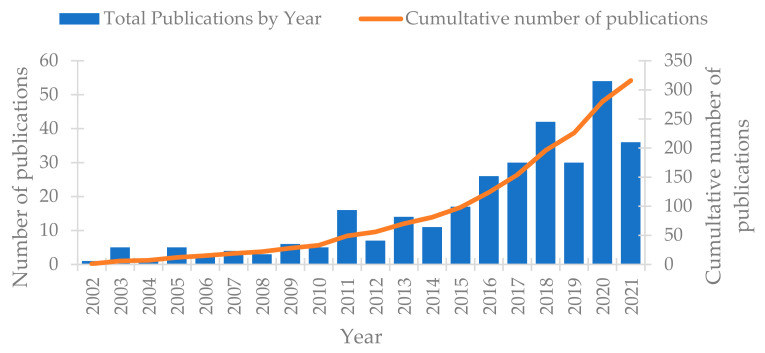
Papers published yearly until October 2021, keywords used “Desalination and Pervaporation” (source: Web of Science).

More recently, several works have reported on the use of pervaporation for desalination purposes [7,20,21] and the interest for desalination by pervaporation seems to be confirmed by the growing number of papers published each year (Figure 1). In the same direction, PV is already used at an industrial scale and the achievable salt rejection is, in theory, higher than 99.99% because salts such as NaCl are not volatile in operating conditions. Thus, the PV membrane acts as a physical barrier and can stop the salt. In addition, the PV membranes are also known to be more stable than MD membranes [22]. Up to now, hydrophilic materials such as Polyvinyl alcohol (PVA), Cellulose acetate (CA) and Cellulose tri-acetate CTA have been mostly used for desalination tests because they are more permeable in water and less sensible to fouling problems [10,21,22,23]. Other inorganic and composite membranes can also be used for desalination by pervaporation, such as graphene and mixed-matrix membrane generation [24,25]. 

**Table 1:** Comparison between MD and PV.

	MD	PV
Membrane type	Porous and hydrophobic	Dense or molecular sieving hydrophilic or hydrophobic
Membrane role	Support medium for the vapor liquid interface Do not contribute to separation	Dense layer contributes to separation by interaction with water molecules: - dipole-dipole interactions - hydrogen bonding - ion-dipole interactions
Mechanism	Knudsen diffusion Poiseuille flow (viscous flow), molecular diffusion	Solution-diffusion, Size exclusion Charge exclusion
Main Configurations	Direct contact MD, vacuum MD, sweeping gas MD, air gap MD	Vacuum PV, sweeping gas PV, air gap PV, direct contact PV (thermo-pervaporation)
Membrane material	PP, PVDF, PTFE	PVA, CTA
Applications	Concentration of juice, desalination, crystallization	Dehydration, recovery of organics, desalination
Challenges	Membrane fouling, membrane wetting and scaling, stability of permeation flux	Relatively lower permeation flux Membrane fouling and scaling
References	[26,27,28]	[10,21,29]

For desalination applications, pervaporation membranes should be hydrophilic, as recommended by several authors in a recent review [22]. Since 1970, Loeb and Sourirajan have developed RO membranes based on cellulose acetate polymers [30] and CTA has also been developed for its stability in a large interval of temperatures and pHs. Interestingly, CTA membranes have less chlorine reactivity than polyamide, another material used for developing commercial RO membranes, and, in addition, CTA has a higher resistance to chemical and biological reactions [31,32,33].

In this paper, theoretical and experimental approaches have been used to develop a new hydrophilic/hydrophobic membrane for desalination by PV based on a dense layer of CTA and PVDF porous support. This CTA composite membrane will be compared to the Teflon™ AF2400 membrane prepared in our previous work on hydrophobic/hydrophobic PV membranes. Finally, the new CTA membrane will be tested with real seawater from the Mediterranean Sea.

## 2. Material and Methods

### 2.1. Chemicals

The pristine support used in this study is a porous PVDF (Durapore™, filter GVHP, pore size 0.22 µm, porosity 75%, thickness: 122 µm), purchased from Merck Millipore. A CTA polymer (Mw 99,000–110,000) and dioxane solvent were obtained from Sigma Aldrich, St. Louis, MO, USA. Figure S1 (see Supplementary Data) shows the structure of the PVDF and CTA polymers. The aqueous solutions were prepared with ultra-pure deionized water Milli-Q (conductivity < 3 μS/cm) and the salt used in the feed (NaCl) and the surfactant Sodium dodecyl sulfate (SDS) were obtained from Sigma-Aldrich. The preparation of the membrane based on PVDF with a TeflonTM AF2400 as the top dense layer has already been reported in our previous work [7] using the same PVDF support and Teflon provided by Dupont.

### 2.2. Membrane Preparation

To prepare membranes with different thicknesses, the concentration of the CTA polymer was changed from 1 to 5 wt % in a dioxane solvent. A known amount of CTA was dissolved and stirred in dioxane to obtain homogenous solutions. Then, an ultrasonic bath was used to eliminate the air bubbles. Next, the applicator film (Elcometer 4340 Motorized Film Applicator) calibrated with the desired wet thickness (50 µm in this study) was used to cast the polymer solutions on the surface of the PVDF support. Finally, after evaporation of the solvent, the prepared membranes were dried for several days at room temperature before characterizations and utilization. PVDF pristine support is noted M0 and membranes coated with CTA are noted M1 to M4. It should be noted that a composite membrane with Teflon™ AF2400 (noted Teflon) was prepared using a similar protocol; more details can be found in our preview work [7].

### 2.3. Characterization Techniques

The prepared membranes were characterized by infrared spectroscopy (IR) (BRUKER, ALPHA-P, Billerica, MA, USA) in attenuated total reflection (ATR mode) and by scanning electron microscope (SEM) (JEOL, JSM 6490LV, Tokyo, Japan). The contact angles were measured with the apparatus Data physics Instruments GmbH (DI) Filderstadt, Germany equipped with a goniometer OCA-PSA Drop 8 using the software SCA20. More details on the characterization methods can be found in previous work [34].

### 2.4. Permeation Experiment

The membranes (composite and pristine support) were tested in a direct contact cell [7,34] with 40 cm^2^ as the active area. The centrifuge pumps, temperature and flow rate sensors were provided by RS Components. Both the feed and the permeate solutions were circulated in closed loops (countercurrent configuration) and the flow rates were 1 L/min on the feed side and 0.8 L/min on the permeate side. The temperature of both sides of the membrane cell was fixed and controlled by a heating system at the feed side and a chiller at the permeate side. The conductivity measurements were performed with the bench conductivity meter Jenway 4520, purchased with glass a conductivity probe with ATC (K = 1/cm). A balance (precision 0.01 g) from Sartorius was used to measure the mass of water obtained at the permeate side. All data were recorded by computer using LabVIEW software (2016).

## 3. Results and Discussion

### 3.1. Membrane Characterization

#### 3.1.1. Fourier Transform InfraRed Spectroscopy (FTIR)

**Figure 2:**
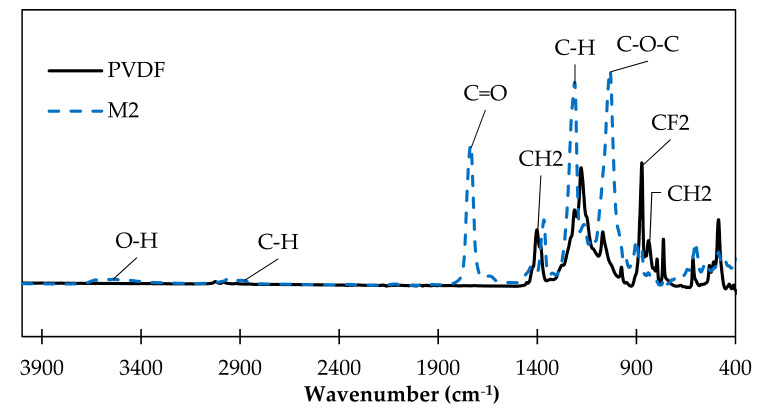
FTIR spectra of pristine PVDF and M2 (PVDF support + CTA) membranes.

The chemical structures of the M0 (pristine PVDF) and M2 (PVDF support + CTA layer) membranes were characterized by FTIR (Figure 2). The characteristic bands of the CF2 groups of the PVDF polymer are clearly identified at 872 cm^−1^, as expected for PVDF [35,36], as well as the CH2 bands at 840, 1400 cm^−1^. All the bands of CTA were also identified: the stretching C=O group at 1733, acetate C-O-C group at 1029 cm^−1^, bending of C-H at 1209, 1420 and 2900 cm^−1^, group C-O at 896 cm^−1^ and stretching of O-H group at 3500 cm^−1^ [37,38].

FTIR analysis showed that the characteristic absorption peaks of pristine PVDF disappeared in the spectra of the coated membrane; this indicates that the whole surface of the PVDF support is totally covered by a CTA layer. Indeed, the absorption peaks characteristic of CTA appeared on the spectra of the coated membrane, such as the C=O stretching which was clearly identified at 1733 cm^−1^. The FTIR results confirm that the surface of the PVDF support was well-coated by the CTA polymer.

#### 3.1.2. Surface Characterizations

The measurement of the contact angle of M0 and top surface of the composite membranes M2 are presented in Table 2, showing, as expected, that the contact angle of PVDF is higher than 90°; this means the surface of the PVDF support is hydrophobic. Conversely, the top surface of the M2 membrane was found to be hydrophilic (contact angle < 90°). These results are in agreement with the values reported in the literature [39,40,41]. The contact angle measurements clearly confirm the modification of the PVDF surface from hydrophobic to hydrophilic; this is due to the CTA dense layer coated on the top surface.

#### 3.1.3. Membrane Morphology

The morphology of coated and uncoated PVDF was examined by SEM, and the corresponding views are shown in Table 3. The porous structure of the pristine PVDF (M0) can be clearly identified both on the top and on the bottom surface, according to the specifications of the commercial support, i.e., a high porosity (70%) and a small pore size (0.22 µm). The top surfaces of all the composite membranes (M1 to M4) are merely different: no pore could be observed, and they seemed to be totally covered by a dense layer of CTA polymer. Different thicknesses of the CTA layer were prepared, and section images clearly show a uniform coating on the surface of the PVDF support, with the dense layer following the topography of the support surface.

### 3.2. Membrane Performance with Synthetic Solution

All experiments were carried out with a direct contact membrane cell (DCM) and the temperature of the feed and the permeate were fixed, respectively, at 50 °C and 20 °C. To evaluate the membrane performances as well as the resistance to the wetting hazard, a specific experimental procedure has been followed with all tested membranes. Once the membrane is well fixed in the cell and the up-and-down stream sides temperature is well stabilized, the experiment starts using pure water in the feed compartment to reach the steady-state flux and to determine the membrane permeance of the pure water. Usually, the conductivity of water is very low, in the range of some µS/cm. After one hour, a concentrated solution of salt is added to obtain a feed with a 3 g/L concentration. Obviously, the feed water conductivity is significantly increased and is stabilized to about 6.5 mS/cm. Note that this value is three orders of magnitude higher than with pure water. After one more hour, the salt concentration is increased up to 10 g/L, leading to a conductivity of about 18.9 mS/cm. Finally, after one more hour, the surfactant SDS is added to give a concentration of 1 g/L, which is a relatively high value. This last part of the experiment with this binary mixture of salt and surfactant was specially designed to give precious information about the wettability resistance of the membrane. Of course, during this experiment, all the operation conditions linked to the flow rate of the feed and permeate, as well as the upstream and downstream temperatures, are kept steady as far as possible.

The advantages of this procedure are to allow the determination of the permeances of water and of the salted solutions with the same membrane sample in a continuous experiment, and to check the occurrence of any defect due to the cell or membrane by controlling, simultaneously, the conductivity of the permeate side.

It is known that the driven force is linked to the difference of temperature between both sides of the membrane, which induces the difference of vapor partial pressures of water between both sides of the membrane. Using Antoine’s law, the vapor partial pressure can be easily determined from the temperature difference (Figure S2: see Supplementary Data).

**Figure 3:**
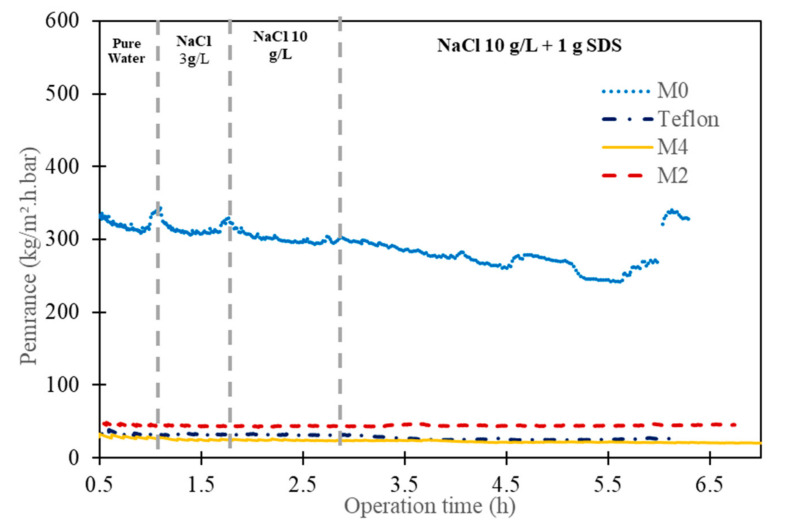
Permeance investigations of pristine PVDF (M0) and composite membranes (M4, M2, Teflon) versus feeds containing pure water, NaCl solutions with and without surfactant SDS.

**Figure 4:**
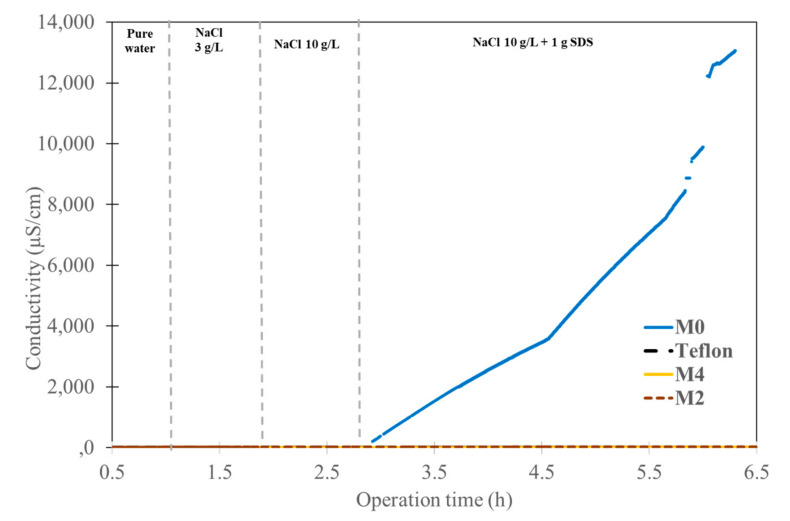
Conductivity investigation of pristine PVDF and composite membranes (M2, M4, Teflon) versus feeds containing pure water, NaCl solutions and NaCl with surfactant SDS.

Figure 3 summarizes the permeance measurements of the M0 and composite membranes (M2, M4, Teflon). The permeance of the porous PVDF (M0) is about 320 kg.m^−2^·h^−1^·bar^−1^ and it slightly decreases with time for water and salt solution conditions; this means the pristine PVDF initially exhibits resistance to wetting and then starts to be partially wetted. Then, the permeance of the PVDF decreases rapidly when the surfactant SDS is added, and it still decreases until the membrane is totally wetted. At this point, the driven force is lost, and the system starts to behave similarly to a microfiltration process. The permeance increases rapidly but without any salt rejection. This explanation is confirmed by the conductivity measurement (Figure 4) when the surfactant is added; the conductivity increases dramatically from a few µS/cm to mS/cm. The same type of observation was reported in the literature [42]. On the other hand, the permeances of the composite membranes that had a dense layer of CTA (hydrophilic) or AF2400 (hydrophobic) were steady whatever the operation conditions, and the salt rejection was still close to 100%, as shown by the very low conductivity during all the experiment.

In addition, the permeance of the M2 (46 kg.m^−2^·h^−1^·bar^−1^) membrane is higher than Teflon (30 kg·m^−2^·h^−1^·bar^−1^), although the dense layer thickness of the M2 is about 4 µm and is about 2 µm for the Teflon membrane. This can be explained by the intrinsic water vapor permeability of the CTA polymer (14,000 Barrer) [43] which is four times higher than that of the AF2400 polymer (4026 Barrer) [44]. Moreover, the second important parameter is the thickness of the dense layer; the M2 membrane with a dense layer thickness of only 4 µm is more permeable than the M4 membrane (thickness ~7 µm; permeance: 29 kg·m^−2^·h^−1^·bar^−1^).

It was observed that when SDS is added, the permeance is slightly decreased but without a change to permeate conductivity. The permeance change can be explained by the change of water activity in the feed side due to the addition of the SDS and salt in the feed side.

Finally, it was found that the procedure used here is a good tool to study the wetting resistance of coated membranes within a short experiment time.

### 3.3. Thickness Effect on Membrane Performance

The thickness of the dense layer is a very important parameter which must be controlled because the transfer matter resistance strongly depends on this parameter. Obviously, a compromise must be found between a low thickness, to favor higher permeance, and the occurrence of defects that might happen with too-low thicknesses. For this reason, the study of the dense layer thickness is important to understand its effect on membrane performance. Liang et al. reported that water permeability of the PVA layer significantly decreases with the thickness of the PVA layer coated on a porous polyacrylonitrile (PAN) [45]. In the same direction, Table 4 shows the experimental data of permeance and thickness for CTA membranes. This Table confirms that, to get a high permeance of PVDF+CTA membranes, a low thickness of CTA dense layer is needed, because the permeance is inversely proportional to the thickness of the dense layer. For example, the dense layer thickness of CTA at about ~1.4 µm gives 115 kg·m^−2^·h^−1^·bar^−1^. However, with a dense layer thickness of 0.5 µm, the permeance should rise up to 390 kg·m^−2^·h^−1^·bar^−1^. This is well within the range of the pristine PVDF (322 kg·m^−2^·h^−1^·bar^−1^).

### 3.4. Raw Seawater Desalination

**Figure 5:**
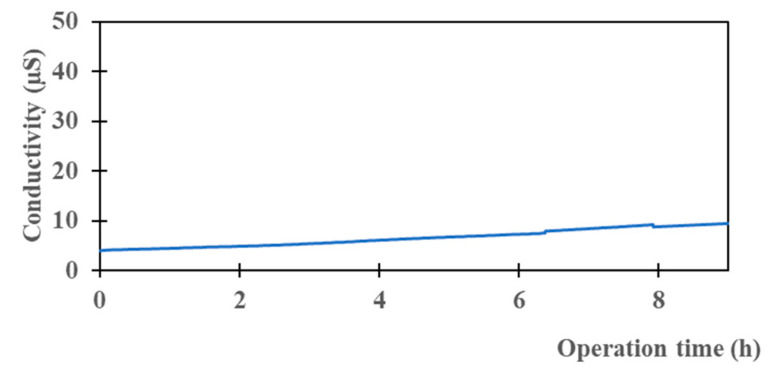
Evolution of permeate conductivity versus time of M2 for seawater.

**Figure 6:**
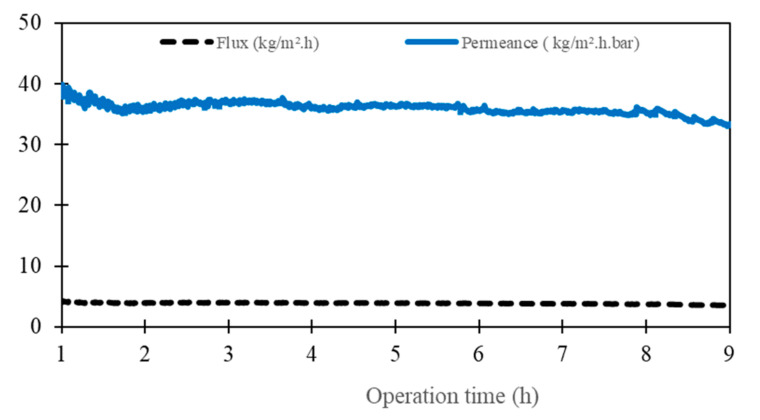
Flux and permeance of M2 for the seawater test.

To evaluate the desalination ability of a composite CTA membrane with real raw conditions, an M2 (~4 µm) membrane was selected and the operating temperatures of the feed and permeate were fixed, respectively, at Tf = 50 °C and Tp = 20 °C. The seawater was collected from the Mediterranean Sea near the Montpellier area in France (GPS: 43°32′26.6″ N 3°58′13.8″ E) and it was used directly without any pretreatment or additives (More information on driving forces (Figure S3) and permeate mass evolution (Figure S4) can be found in Supplementary Data). The conductivity, TDS and the total salinity of the feed and the permeate were measured (Table 5). The salt rejection was higher than 99.5% and the conductivity did not change (between 6 and 10 µS/cm) (Figure 5). The flux was about 4 kg·m^−2^·h−^1^ and the permeance was about 37 kg·m^−2^·h^−1^·bar^−1^ (Figure 6). As expected, the permeance of seawater is lower than the permeance of pure water (46 kg·m^−2^·h^−1^·bar^−1^). This can be explained by the presence of various salts in the seawater which affect water activity.

### 3.5. Theoretical Prediction and Comparison with Literature

**Figure 7:**
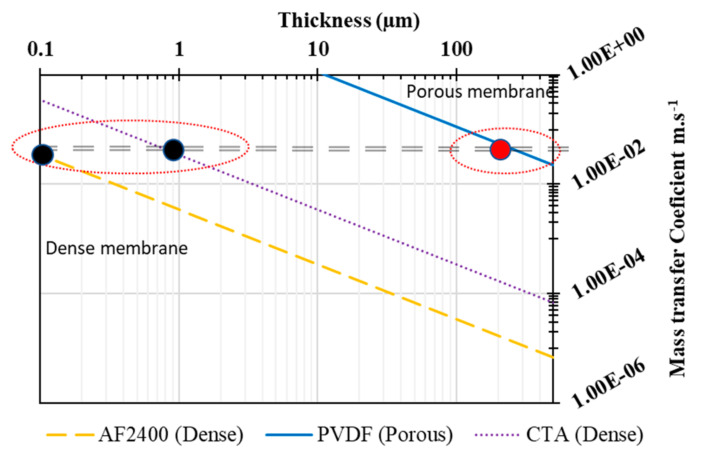
Prediction of the mass transfer coefficients (km) of various dense layers (dashed lines) of Teflon AF2400 or CTA and porous PVDF membranes (solid line). The km of the PVDF support (5.6 × 10^−2^ m·s^−1^ for a thickness of 200 μm) was calculated from our experimental data and the km of the dense layers from the literature data [43,44].

The global mass transfer coefficients of pristine and composite membranes were easily calculated from previous publications [7,44,46] and Figure 7 presents these results. It is clear that the mass transfer coefficient depends on the thickness and the nature of the polymer material. When the thickness decreases, the mass transfer coefficient increases. In addition, it is known that the intrinsic permeability changes from polymer to the other. For example, the water permeability of a CTA polymer is much higher than that of an AF2400 polymer. In Figure 7, the mass transfer coefficient of porous membrane with about 200 µm (red circle), is equal to CTA dense membrane with about 1 µm thickness (black circles) and it is equal to AF2400 dense membrane with about 0.1 µm (black circle).

It should be noted that the mass transfer coefficient of the PVDF porous membrane and dense membranes CTA, AF2400 was calculated based on the theoretical model of resistance in series to consider both the porous and the dense polymer thicknesses.

Table 6 present a simulation of the permeation and mass transfer coefficient of different composite membranes (hydrophilic or hydrophobic dense layer) and the equation (1) from our previous study [7] was used to calculate the mass transfer coefficient for 0.1 µm as the thickness of the active layer. It is known that the permeance is inversely proportional to the dense layer thickness; therefore, the permeance can increase rapidly if the dense layer thickness decreases.

Based on this calculation, it is possible to get the same mass transfer coefficient with a porous and dense membrane if two conditions are verified: (i) the thickness of the dense layer must be as low as possible and (ii) the dense polymer must also have an intrinsically high permeability. The experimental data confirm these theoretical results. Therefore, it is possible to use a coating dense layer to avoid wetting of the porous membrane during seawater desalination. This solution definitively prevents the entrance of liquid water into the pores, as observed for porous membrane in membrane distillation, and a very small dense layer thickness can give an acceptable permeance.

Moreover, Table 7 shows data from the literature compared with our experimental data for real seawater. It is difficult to compare these data because there are many different parameters, such as concentration of salt, temperature, thickness of membrane…However, the flux depends on the thickness and type of polymer that confirms our experimental strategy. Recently, Ali et al. are reported on a successful new protocol to make an ultra-selective defect-free thin film composite membrane for desalination and gas separation by interfacial polymerization. The apparent thickness was between 68 and 500 nm and the protocol was an industrially scalable roll-to-roll method. They also reported the existence of a commercial NF270 membrane with an apparent thickness of 45 nm [47]. It is interesting to note that if the thickness of the dense layer can be limited to 100 nm, the flux would reach 40 kg/(m^2^·h). Considering the actual development of coating techniques, it seems feasible to reach this range of thickness at an industrial scale.

## 4. Conclusions

The main goal of this study was to use theoretical and experimental approaches to develop a specific membrane for desalination by pervaporation, and hence, to open a new gate for the potential applications of saline water such as seawater and RO brine rejection. Indeed, the transfer mechanism by PV can lead to a much higher efficiency than the RO process for hypersaline solutions.

The main points of this work are the following:

The experimental approach led to good composite membranes, using PVDF as porous support and CTA as a thin, dense coating layer. The composite membranes exhibited a good compatibility between the CTA polymer and PVDF support without any intrusion into the porous hydrophobic support (SEM images).

Tested in the same conditions with pure water, saline water and even with a surfactant (SDS), the composite membranes had steady desalination properties. Compared to the pristine PVDF membrane, no wetting occurred, and in addition, a high salt rejection (near to 100%) could be achieved.

Furthermore, with real seawater from the Mediterranean Sea without any pretreatment, the permeance of the PVDF/CTA composite was tested for 9 h, giving rise to results close to the permeance of pure water. In addition, the salt rejection remained very high (99.5%).

Finally, it can be said that this type of hydrophobic/hydrophilic composite membrane looks promising to develop efficient membranes able to achieve the desalination of seawater and of hypersaline water feeds by pervaporation.

## Figures and Tables

**Table 2:** Contact angle of PVDF before (M0) and after surface modification with CTA(M2).

Membrane	Contact Angle °	Deviation	Image
M0	126	2.16	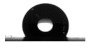
M2	57	3.28	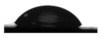

**Table 3:** SEM images of pristine PVDF (M0) and PVDF+CTA composites membranes (M1 to M4).

Membrane	Dense Layer Thickness (µm ± 0.5 µm)	Cross-Section	Top Surface	Bottom Surface
M0	without coating	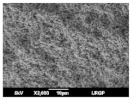	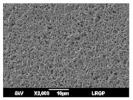	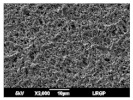
M1	1.4	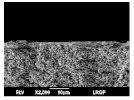	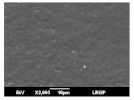	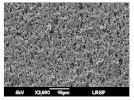
M2	3.8	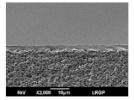	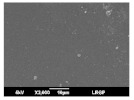	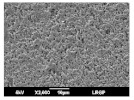
M3	4.1	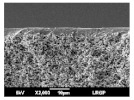	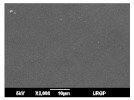	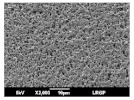
M4	6.8	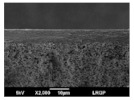	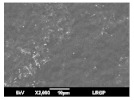	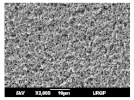

**Table 4:** Variation of permeance versus thickness of dense layer.

Membrane	Dense Layer Thickness (µm)	Permeance (kg.m^−2^.h^−1^.bar^−1^)
M1	1.4	115
M2	3.8	46
M3	4.1	43
M4	6.8	29

**Table 5:** Characterization of the feed and permeate solutions of seawater desalination by the M2 membrane (measurements at 19 °C).

	Feed	Permeate	Retention %
Salinity g/L	38.3	0.3	99.2
Conductivity (mS/cm)	56.3	0.009	99.9
TDS (g/L)	40.8	0.005	99.9

**Table 6:** Calculation of the mass transfer coefficients and water permeance of the composite membranes using the experimental PVDF support permeance and the permeability of the dense top layer of CTA or Teflon AF2400.

	Support	Prediction for Composite Membranes ^b^	
	PVDF (122 µm) ^a^	CTA (0.1 µm) ^b^	Teflon (0.1 µm) ^b^
Mass transfer coefficient (m/s)	1.21 × 10^−1^	6.1 × 10^−2^	2.7 × 10^−2^
Water permeance (kg·m^−2^·h^−1^·bar^−1^)	323.47	163	72

^a^ Calculated from the experimental data. ^b^ Calculated according the Equation (2) from previous work [7] using the literature permeability data; the conversion factor permeability from Barrer to (mol·m)/(m^2^·s·Pa) is 3.34 × 10^−16^·s.

**Table 7:** Comparison of different membranes.

Membrane	NaCl (ppm)	Feed Temperature (°C)	Thickness (µm)	Flux kg/(m^2^·h)	Rejection (%)	Reference
CTA	100,000	50	10	2.3	99	[48]
Polyester	35,000	70	20–25	5.97–3.45	99.7	[49]
PVA/MA	30,000	70	0.1	7.4	99.9	[50]
Polyether amide	35,000	46–82	40	0.2	99.99	[51]
S-PVA/PAN	35,000	70	0.8	46.3	99.5	[45]
CTA/PVDF	Seawater 37,300	50	4	4	99.5	this study

## Supplementary Materials

The following supporting information can be downloaded at: https://www.mdpi.com/article/10.3390/membranes13060599/s1, Figure S1: Chemical structure of polymers: (a) PVDF and (b) CTA. Figure S2: The evolution of driven force for pristine PVDF and composites membranes in versus time. Figure S3: the driving force Delta T and Delta P in versus time of M2 for seawater test. Figure S4: Evolution of permeate mass with time of M2 for raw seawater.

## References

[1] Bremere I., Kennedy M., Stikker A., Schippers J. (2001). How water scarcity will effect the growth in the desalination market in the coming 25 years. Desalination.

[2] Feria-Díaz J., Maria Cristina L.-M., Sandoval-Herazo L., Correa Mahecha F., Rodriguez Miranda J. (2021). Commercial Thermal Technologies for Desalination of Water from Renewable Energies: A State of the Art Review. Processes.

[3] Curto D., Franzitta V., Guercio A. (2021). A Review of the Water Desalination Technologies. Appl. Sci..

[4] Jones E., Qadir M., van Vliet M.T., Smakhtin V., Kang S.-M. (2018). The state of desalination and brine production: A global outlook. Sci. Total Environ..

[5] Imbrogno J., Keating J.J., Kilduff J., Belfort G. (2017). Critical aspects of RO desalination: A combination strategy. Desalination.

[6] McGinnis R.L., Elimelech M. (2008). Global Challenges in Energy and Water Supply: The Promise of Engineered Osmosis. Environ. Sci. Technol..

[7] Eljaddi T., Mendez D.L.M., Favre E., Roizard D. (2021). Development of new pervaporation composite membranes for desalination: Theoretical and experimental investigations. Desalination.

[8] Elsaid K., Sayed E.T., Abdelkareem M.A., Baroutaji A., Olabi A. (2020). Environmental impact of desalination processes: Mitigation and control strategies. Sci. Total Environ..

[9] Drioli E., Ali A., Macedonio F. (2015). Membrane distillation: Recent developments and perspectives. Desalination.

[10] Wang L., Wang Y., Wu L., Wei G. (2020). Fabrication, Properties, Performances, and Separation Application of Polymeric Pervaporation Membranes: A Review. Polymers.

[11] Golubev G., Eremeev I., Makaev S., Shalygin M., Vasilevsky V., He T., Drioli E., Volkov A. (2021). Thin-film distillation coupled with membrane condenser for brine solutions concentration. Desalination.

[12] Bindels M., Carvalho J., Gonzalez C.B., Brand N., Nelemans B. (2020). Techno-economic assessment of seawater reverse osmosis (SWRO) brine treatment with air gap membrane distillation (AGMD). Desalination.

[13] Kaminski W., Marszalek J., Tomczak E. (2018). Water desalination by pervaporation–Comparison of energy consumption. Desalination.

[14] Franken A., Nolten J., Mulder M., Bargeman D., Smolders C. (1987). Wetting criteria for the applicability of membrane distillation. J. Membr. Sci..

[15] Lawson K.W., Lloyd D.R. (1997). Membrane distillation. J. Membr. Sci..

[16] Essalhi M., Khayet M. (2015). Membrane Distillation (MD). Progress in Filtration and Separation.

[17] Horseman T., Yin Y., Christie K.S., Wang Z., Tong T., Lin S. (2020). Wetting, Scaling, and Fouling in Membrane Distillation: State-of-the-Art Insights on Fundamental Mechanisms and Mitigation Strategies. ACS ES&T Eng..

[18] Jyoti G., Keshav A., Anandkumar J. (2015). Review on Pervaporation: Theory, Membrane Performance, and Application to Intensification of Esterification Reaction. J. Eng..

[19] Cannilla C., Bonura G., Frusteri F. (2017). Potential of Pervaporation and Vapor Separation with Water Selective Membranes for an Optimized Production of Biofuels—A Review. Catalysts.

[20] Xie Z., Li N., Wang Q., Bolto B. (2018). Desalination by pervaporation. Emerg. Technol. Sustain. Desalin. Handb..

[21] Wang Q., Li N., Bolto B., Hoang M., Xie Z. (2016). Desalination by pervaporation: A review. Desalination.

[22] Castro-Muñoz R. (2020). Breakthroughs on tailoring pervaporation membranes for water desalination: A review. Water Res..

[23] Zhao P., Xue Y., Zhang R., Cao B., Li P. (2020). Fabrication of pervaporation desalination membranes with excellent chemical resistance for chemical washing. J. Membr. Sci..

[24] Guan K., Liu G., Matsuyama H., Jin W. (2020). Graphene-based membranes for pervaporation processes. Chin. J. Chem. Eng..

[25] Yang G., Xie Z., Cran M., Wu C., Gray S. (2020). Dimensional Nanofillers in Mixed Matrix Membranes for Pervaporation Separations: A Review. Membranes.

[26] Warsinger D.M., Swaminathan J., Guillen-Burrieza E., Arafat H.A., Lienhard V.J.H. (2015). Scaling and fouling in membrane distillation for desalination applications: A review. Desalination.

[27] Yao M., Tijing L.D., Naidu G., Kim S.-H., Matsuyama H., Fane A.G., Shon H.K. (2020). A review of membrane wettability for the treatment of saline water deploying membrane distillation. Desalination.

[28] Eykens L., De Sitter K., Dotremont C., Pinoy L., Van der Bruggen B. (2017). Membrane synthesis for membrane distillation: A review. Sep. Purif. Technol..

[29] Nagy E. (2019). Pervaporation. Basic Equations Mass Transport through a Membrane Layer.

[30] You M., Yin J., Sun R., Cao X., Meng J. (2018). Water/salt transport properties of organic/inorganic hybrid films based on cellulose triacetate. J. Membr. Sci..

[31] Nakao T., Miura Y., Furuichi K., Yasukawa M. (2021). Cellulose Triacetate (CTA) Hollow-Fiber (HF) Membranes for Sustainable Seawater Desalination: A Review. Membranes.

[32] Lee K.P., Arnot T.C., Mattia D. (2011). A review of reverse osmosis membrane materials for desalination—Development to date and future potential. J. Membr. Sci..

[33] Nguyen T.P.N., Jun B.-M., Kwon Y.-N. (2017). The chlorination mechanism of integrally asymmetric cellulose triacetate (CTA)-based and thin film composite polyamide-based forward osmosis membrane. J. Membr. Sci..

[34] Eljaddi T., Mendez D.L.M., Favre E., Roizard D. (2021). Development of new pervaporation composite membranes for desalination: Membrane characterizations and experimental permeation data. Data Brief.

[35] Azmi R., Goh P., Ismail A., Lau W., Ng B., Othman N., Noor A., Yusoff M. (2015). Deacidification of crude palm oil using PVA-crosslinked PVDF membrane. J. Food Eng..

[36] He F., Luo B., Yuan S., Liang B., Choong C., Pehkonen S.O. (2013). PVDF film tethered with RGD-click-poly(glycidyl methacrylate) brushes by combination of direct surface-initiated ATRP and click chemistry for improved cytocompatibility. RSC Adv..

[37] Kebiche-Senhadji O., Bey S., Clarizia G., Mansouri L., Benamor M. (2011). Gas permeation behavior of CTA polymer inclusion membrane (PIM) containing an acidic carrier for metal recovery (DEHPA). Sep. Purif. Technol..

[38] Fei P., Liao L., Cheng B., Song J. (2017). Quantitative analysis of cellulose acetate with a high degree of substitution by FTIR and its application. Anal. Methods.

[39] Khongnakorn W., Bootluck W., Youravong W. (2014). Surface Modification of CTA-FO Membrane by CO_2_ Plasma Treatment. J. Teknol..

[40] Ghaemi N., Madaeni S.S., Alizadeh A., Daraei P., Zinatizadeh A.A., Rahimpour F. (2012). Separation of nitrophenols using cellulose acetate nanofiltration membrane: Influence of surfactant additives. Sep. Purif. Technol..

[41] Chen Y., Tian M., Li X., Wang Y., An A.K., Fang J., He T. (2017). Anti-wetting behavior of negatively charged superhydrophobic PVDF membranes in direct contact membrane distillation of emulsified wastewaters. J. Membr. Sci..

[42] Rezaei M., Samhaber W. (2016). Wetting behaviour of superhydrophobic membranes coated with nanoparticles in membrane distillation. Chem. Eng. Trans..

[43] Chen G.Q., Kanehashi S., Doherty C.M., Hill A.J., Kentish S.E. (2015). Water vapor permeation through cellulose acetate membranes and its impact upon membrane separation performance for natural gas purification. J. Membr. Sci..

[44] Mendez D.L.M., Castel C., Lemaitre C., Favre E. (2018). Membrane distillation (MD) processes for water desalination applications. Can dense selfstanding membranes compete with microporous hydrophobic materials?. Chem. Eng. Sci..

[45] Liang B., Li Q., Cao B., Li P. (2018). Water permeance, permeability and desalination properties of the sulfonic acid functionalized composite pervaporation membranes. Desalination.

[46] Makhloufi C., Lasseuguette E., Remigy J.C., Belaissaoui B., Roizard D., Favre E. (2014). Ammonia based CO_2_ capture process using hollow fiber membrane contactors. J. Membr. Sci..

[47] Ali Z., Wang Y., Ogieglo W., Pacheco F., Vovusha H., Han Y., Pinnau I. (2020). Gas separation and water desalination performance of defect-free interfacially polymerized para-linked polyamide thin-film composite membranes. J. Membr. Sci..

[48] Huth E., Muthu S., Ruff L., Brant J.A. (2014). Feasibility assessment of pervaporation for desalinating high-salinity brines. J. Water Reuse Desalination.

[49] Naim M., Elewa M., El-Shafei A., Moneer A. (2015). Desalination of simulated seawater by purge-air pervaporation using an innovative fabricated membrane. Water Sci. Technol..

[50] Chaudhri S.G., Rajai B.H., Singh P.S. (2015). Preparation of ultra-thin poly(vinyl alcohol) membranes supported on polysulfone hollow fiber and their application for production of pure water from seawater. Desalination.

[51] Zwijnenberg H.J., Koops G., Wessling M. (2005). Solar driven membrane pervaporation for desalination processes. J. Membr. Sci..

