# Supplementary material for: Design and Preparation a New Composite Hydrophilic/Hydrophobic Membrane for Desalination by Pervaporation
Source: Membranes (Basel). 2023 Jun 13;13(6):599. doi: 10.3390/membranes13060599 (PMC10301471; doi:10.3390/membranes13060599)
Supplement: Supplementary file 1 [file membranes-13-00599-s001.zip › membranes-2391949-supplementary.pdf]

Supplementary Data

Design and preparation a new composite hydrophilic/hydrophobic membrane for desalination by pervaporation

T. Eljaddi*, E. Favre*, D. Roizard*

* LRGP—Laboratoire Réactions et Génie des Procédés, UMR 7274, 54001 Nancy, France.

1. Polymer structure:

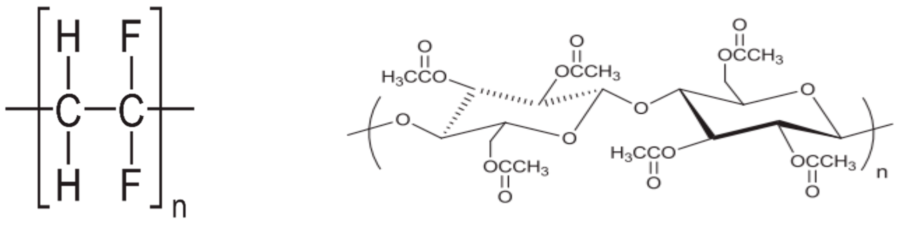

a ***b***

Figure S1: Chemical structure of polymers: a) PVDF and b) CTA

2. Membrane performance test:

The **Figure S2** indicates that all driven forces (delta P) for all membranes (Pristine PVDF, and PVDF+CTA (M2 and M4)) were constant all along the 3 hours, i.e. corresponding to the three set of conditions (pure water, 3g/L and 10g/L salt concentration). After this 3h period, just after the addition of the surfactant, surprisingly, the driven force of composites membranes is still constant while the driven force of the pristine PVDF membrane tends to decrease steadily. Hence it means that the temperature difference must also be decreasing. Thus, this is an indicator that wetting it is occurring and it was confirmed by permanence and conductivity measurements recorded during experiments.

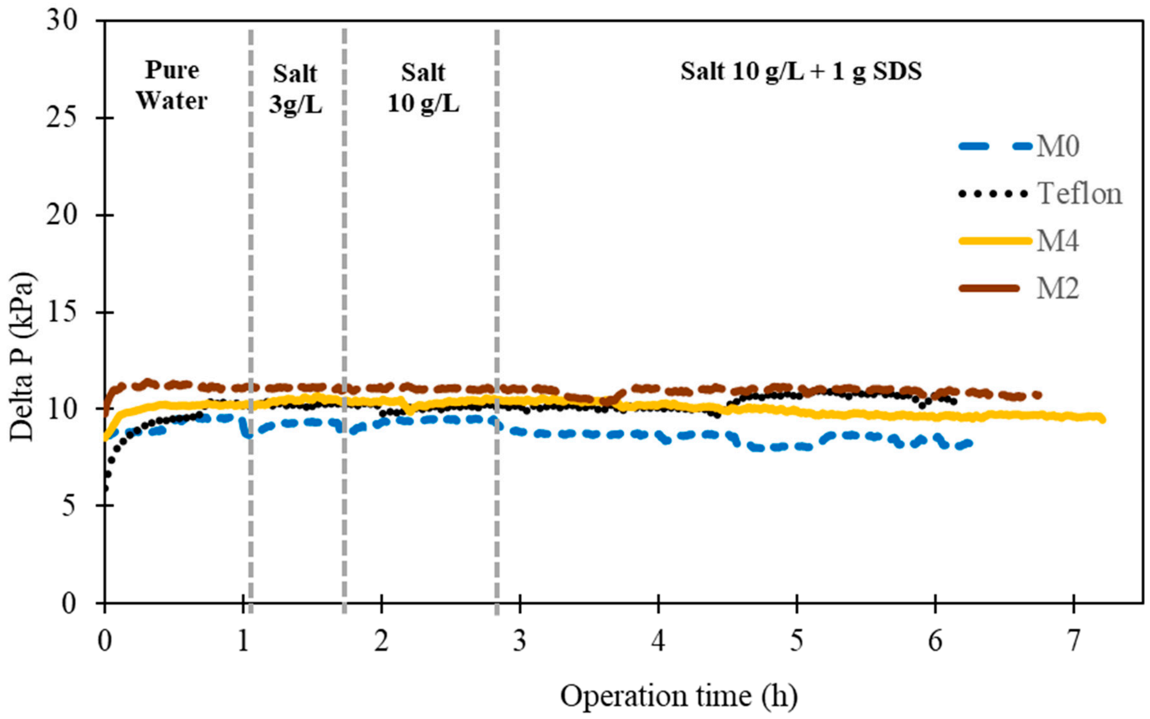

Figure S2: The evolution of driven force for pristine PVDF and composites membranes in versus time

3. Seawater test:

. The driven force DeltaT or DeltaP was registered was still constant (respectively 31 °C and 11 kPa) while the duration of experiments (about 9 hours) (**Figure S3**), the permeate mass follows a linear law ($Mass_{permeate}$ =15.133×t with a correlation factor $R^2 \approx 0.999$) (**Figure S4**)

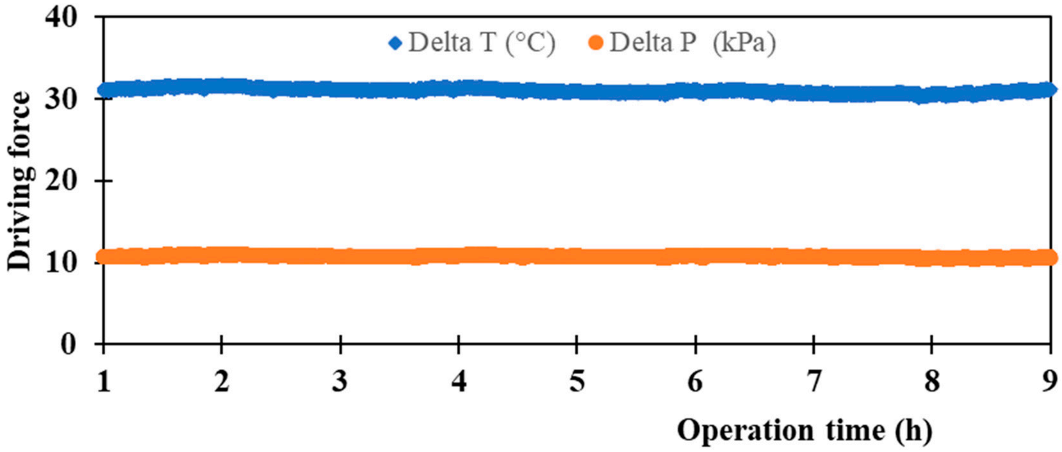

Figure S3: the driving force Delta T and Delta P in versus time of M2 for seawater test

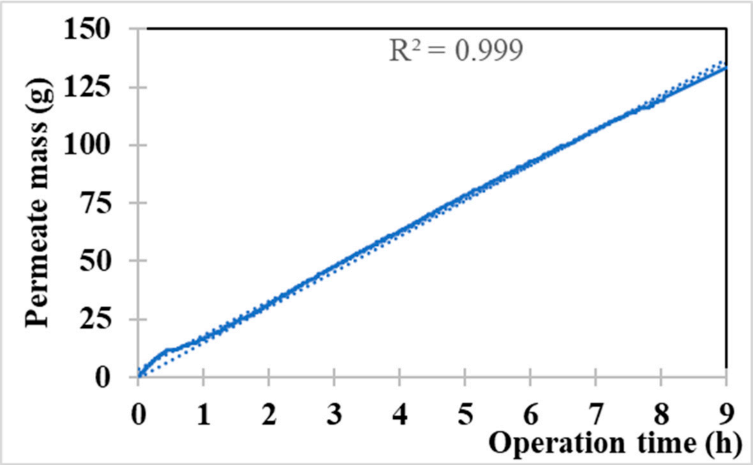

FigureS4: Evolution of permeate mass with time of M2 for raw seawater.
